# Oncoplastic and Reconstructive Surgery of the Breast

**DOI:** 10.1038/sj.bjc.6602351

**Published:** 2005-01-25

**Authors:** M Hamdi

**Affiliations:** 1 Gent University Hospital, Gent, Belgium

This textbook represents a significant contribution to the body of literature dedicated to oncoplastic surgery. While there are several textbooks on breast reconstruction, there are but a few that focus on this unique field.

The first chapter summarises the definition of the specialist breast surgeon in three categories: one who is able to perform tumour resection and all kinds of breast reconstruction; one who is able to do only implant reconstruction with or without a latissimus dorsi flap; and finally a team where the tumour resection is performed by the oncological surgeon and the reconstruction is carried out by the plastic surgeon. I support the last scenario in which everyone works in his or her own specialty. Reconstructive surgery is not simply filling a defect, but it encompasses a wide knowledge of tissue handling, perfusion and healing, in addition to achieving acceptable aesthetic results. Moreover, there is a concern that a plastic surgeon performing the tumour excision might compromise the oncological guidelines by minimising the defect in order to obtain a better aesthetic outcome. Individual cases should be discussed at multidisciplinary team meetings and surgical treatment can then be planned easily and included within protocols.

The first section on mastectomy and implant reconstruction provides a comprehensive review of the evolution of implants. New or modified approaches to the mastectomy are described and are very useful to decrease postoperative complications. Recent developments in implant shapes, envelope and gel structure, such as the use of anatomical implants with cohesive gel, are reported. Surgical techniques are well illustrated, in particular, preserving or creating the inframammary fold.

The second section covers flap surgery for postmastectomy reconstruction. The authors present a basic and objective approach regarding patient selection, surgical techniques and results. Comprehensive steps are described to help improve the results. It is clear that the authors are among the pioneers in this field. The chapter on free flaps was more difficult to read. The author attempts to cram too much information on this field, which could necessitate an entire book. Moreover, only the abdomen was cited as a donor site for free flaps. Other donor sites such as the gluteal region or thigh are not discussed, possibly due to a lack of experience. However, the weakest contribution is the commentary on this chapter, which I feel is presented with little experience. The authors have naively criticised perforator flaps, which include a revolutionary concept in flap surgery, namely, reduction of donor site morbidity to practically nil. The authors give a false impression that this method is associated with higher donor site morbidity than the standard free flap.

The third section is on breast reconstruction after conservative surgery. Personally, I consider this the best part of the book because it summarises the abundant experience of the Milan department. The combination of a quardantectomy with an immediate partial breast reconstruction has been considered as a decisive stage in the evolution of breast cancer surgery. This combination, known as ‘oncoplastic surgery,’ allows a wider resection of the tumour with safe margins together with the advantages of immediate breast reconstruction using supple nonirradiated tissue. This achieves both ultimate goals: adequate local control of the disease and good aesthetic results. The authors describe their technique of using glandular flaps raised on the breast itself for partial reconstruction in combination with contralateral breast remodeling to obtain better symmetry. However, partial breast reconstruction with regional flaps is missing from this section. By using this technique pedicled flaps harvested from the back, such as a latissimus dorsi or a perforator flap, can accomplish partial breast reconstruction without altering the contralateral breast.

In the fourth section of the book, the authors provide separate chapters on psychological issues, chronic pain and rehabilitation, all of which are extremely important topics. The most interesting section is on the biomechanical aspects of breast reconstruction with myocutaneous flaps. The authors show a large body of data regarding biomechanical sequelae of shoulder balance, gait, joint limitation and instabilities, and chronic deteriorations following reconstruction using skin-muscle flaps. They propose a preoperative assessment of posture and muscle synergy in order to choose the optimal method for reconstruction.

Obviously, sparing the muscle by harvesting a flap that consists of skin and fat only, such as is the case with a perforator flap, will prevent or reduce such sequelae to a minimum.

The last chapters deal with patient expectations, contraindications to reconstructive surgery and some other psychological aspects of breast reconstruction. These chapters are very useful and bring something new to surgical textbooks that usually deal more with anatomy, drawings, operative techniques and physical complications. I support the belief that the inclusion of a rehabilitation unit is advantageous in every breast unit.

The book does however give little information on flap surgery, and in particular on free flap surgery, as might be expected of a single volume. I also feel the text would be more concise if it was limited to only oncoplastic surgery.

Overall, the entire group of authors is to be congratulated on the creation of a new textbook in the specialty of oncoplastic surgery with new sections on psychological management of patients with breast reconstruction

